# Reply to von Bünau et al. Comment on “Depoorter, L.; Vandenplas, Y. Probiotics in Pediatrics. A Review and Practical Guide. *Nutrients* 2021, *13*, 2176”

**DOI:** 10.3390/nu14040725

**Published:** 2022-02-09

**Authors:** Yvan Vandenplas, Leontien Depoorter

**Affiliations:** KidZ Health Castle, UZ Brussel, Vrije Universiteit Brussel, 1090 Brussels, Belgium; leontien.depoorter@uzbrussel.be

We agree with Prof. Stange and Erhardt for their comment on our paper on Probiotics in Pediatrics [1], regarding our statement “In adults, several studies showed a beneficial effect of *E. coli* Nissle 1917 compared to standard treatment with mesalazine alone in maintaining remission of the disease [1,2]. Again, these results were not confirmed by any RCT [3].” As Prof. Stange and Erhardt claim, we reported that there are trials with *E. coli* Nissle 1917 in adults. However, there are—to the best of our knowledge—no randomized controlled trials in children. Therefore, we wrote “Again, these studies were not confirmed by any randomized controlled trial” (in children, since the manuscript is about probiotics in pediatrics). So, while there is some evidence in adults, there are no data in pediatrics. 
